# Anti-β2 glycoprotein domain 1 antibody as a diagnostic marker for antiphospholipid syndrome and a predictor of thrombosis: a systematic review and meta-analysis

**DOI:** 10.3389/fimmu.2025.1541165

**Published:** 2025-04-23

**Authors:** Linhui Li, Jian Chen, Jing Feng, Hong Zhao, Xiaojuan Liu, Yongmei Jiang

**Affiliations:** ^1^ Department of Laboratory Medicine, West China Second University Hospital, Sichuan University, Chengdu, China; ^2^ Key Laboratory of Birth Defects and Related Diseases of Women and Children, Ministry of Education, Sichuan University, Chengdu, China

**Keywords:** antiphospholipid syndrome, anti-β2 glycoprotein I domain 1 antibody, thrombosis, meta-analysis, diagnostic accuracy

## Abstract

Anti-β2 glycoprotein I domain 1 (anti-β2GPI-D1) antibodies have shown promise as diagnostic and prognostic markers for antiphospholipid syndrome (APS), but their clinical significance remains uncertain. This systematic review and meta-analysis evaluated the diagnostic accuracy of anti-β2GPI-D1 for APS and its association with thrombotic risk. A comprehensive search was conducted across PubMed, Web of Science, and Embase up to July 18, 2024. Eighteen studies (2,060 APS patients and 3,013 controls) were included in the diagnostic analysis, revealing a pooled sensitivity of 52% (95% CI 46%-58%) and specificity of 95% (95% CI 88%-98%). Anti-β2GPI-D1 demonstrated strong diagnostic value in distinguishing APS from other autoimmune diseases and healthy individuals, though its utility in differentiating APS from aPL carriers was limited. Additionally, five prospective cohort studies (210 APS patients, 430 aPL carriers, and 42 SLE patients) showed that anti-β2GPI-D1 was associated with an increased risk of thrombosis (pooled RR 1.75, 95% CI 1.07-2.87). Our findings suggest that anti-β2GPI-D1 offers high specificity and moderate sensitivity for APS diagnosis and may serve as a predictor of thrombosis.

## Introduction

Antiphospholipid syndrome (APS) is a systemic autoimmune disorder characterized by recurrent thrombosis and/or pregnancy morbidity, along with the persistent presence of antiphospholipid antibodies (aPLs) (1). The current classification criteria for APS involve three aPL tests: lupus anticoagulant (LA), anticardiolipin antibodies (aCL), and anti-β2 glycoprotein I antibodies (anti-β2GPI) (1). Among these aPLs, anti-β2GPI has been widely recognized as the major pathogenic subset in both *in vitro* and animal experiments (2).

Given its recognized pathogenic role, anti-β2GPI has been shown to play a significant role in the development of thrombosis and pregnancy morbidity (3–5). However, its association with specific clinical manifestations remains controversial. For example, a meta-analysis by Reynaud et al. indicated that anti-β2GPI is associated with an increased risk of arterial events, but not with venous thrombosis (6). Another meta-analysis of prospective studies reported that the presence of anti-β2GPI shows only a weak independent association with thrombosis and an inconsistent association with obstetric complications (7).

**Figure 1:**
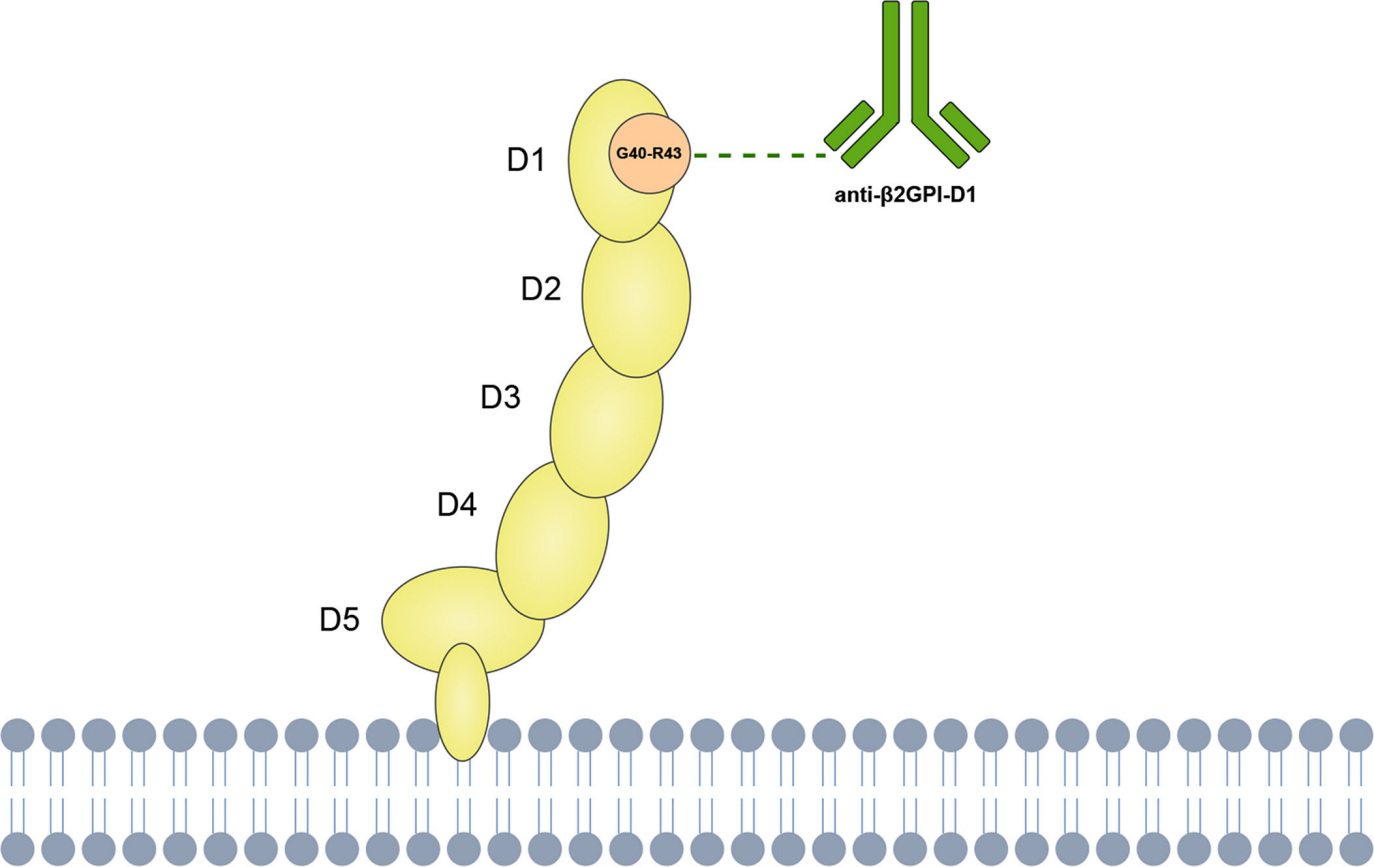
Schematic representation of anti-β2GPI-D1 binding to the Gly40–Arg43 epitope on domain 1 of β2GPI.

This variability in clinical outcomes may be partly explained by the molecular structure of β2GPI, which presents multiple antigenic sites targeted by different autoantibodies (8). β2GPI is a plasma protein composed of five homologous domains (D1-D5), each of which has been identified as a target for anti-β2GPI (9, 10). Among these, the glycine 40-arginine 43 epitope on domain 1 has been highlighted by experimental evidence as the most relevant antigenic target in APS pathogenesis (**Figure 1**). *In vivo* experiments have demonstrated that antibodies against β2GPI, particularly those targeting domain 1, can induce thrombotic and obstetric complications (11, 12). Moreover, treatment with recombinant D1 peptide has been found to inhibit the induction of thrombosis in mouse models (13). Subsequent studies have indicated that anti-β2GPI-D1 is strongly associated with vascular thrombosis and, to a lesser extent, with obstetric complications in APS patients (14, 15). Furthermore, high frequencies and titers of anti-β2GPI-D1 have been identified in patients with triple aPL positivity, suggesting their potential in risk stratification of APS (16, 17). Importantly, anti-β2GPI-D1 antibodies have also shown high specificity and positive predictive value for the diagnosis of APS (16, 18, 19). These studies support the significant role of anti-β2GPI-D1 antibodies in both the pathogenesis and diagnosis of APS. In contrast, studies targeting other domains, such as domain 4/5, have not shown significant involvement in APS-related complications (20, 21). In addition, anti-β2GPI-D1 antibodies, along with other non-criteria aPLs, such as anti-phosphatidylserine/prothrombin (anti-PS/PT) antibodies, have shown promising clinical utility in identifying patients with APS and improving risk stratification. For example, previous studies have reported that anti-PS/PT antibodies coexist with anti-β2GPI-D1 antibodies in approximately 33~41% of APS patients (18, 22). Importantly, the combined positivity of anti-β2GPI-D1 and anti-PS/PT demonstrates a high positive predictive value for APS diagnosis and effectively identifies patients at higher thrombotic risk (18, 22–24).

Despite the growing interest in integrating anti-β2GPI-D1 testing into clinical practice, its clinical utility remains a matter of debate. The clinical value of anti-β2GPI-D1 antibodies, particularly in the diagnosis and prognosis of APS, has yet to be fully clarified. In this study, we conducted a systematic review and meta-analysis of published data to evaluate the diagnostic accuracy of anti-β2GPI-D1 in identifying patients with APS. Furthermore, we sought to examine the risk of thrombosis associated with anti-β2GPI-D1 based on data derived from prospective studies.

## Materials and methods

The methodology of this systematic review and meta-analysis was in accordance with the PRISMA-DTA and PRISMA guidelines (25, 26). The study protocol was pre-registered in the PROSPERO international prospective register of systematic reviews (CRD42024599206).

### Search strategy

A comprehensive search was performed using the Pubmed, Web of Science and Embase databases from inception to July 18, 2024. The search strategy included the following keywords and subject terms: (“beta 2-glycoprotein I”[MeSH Terms] OR “beta 2-glycoprotein I” [All Fields] OR “beta 2-glycoprotein 1”[All Fields]) AND domain [All Fields].

### Study selection

All search records were imported into EndNote X21 software, and duplicates were removed both automatically and manually. Two investigators (LL and JC) independently screened all titles and abstracts for potential relevance. Potentially relevant studies were reviewed in full text according to the following eligibility criteria. Any disagreements between the two independent investigators were resolved by consensus.

### Eligibility criteria

The inclusion criteria for the meta-analysis on diagnostic accuracy were as follows (1): observational studies that included populations of both APS patients and non-APS controls (2); the diagnosis of APS was established according to the laboratory and clinical criteria applicable at the time of the study, namely the Sapporo Criteria (27), Sydney Criteria (28), or 2023 ACR/EULAR Criteria (29) (3); studies that measured anti-β2GPI-D1 in serum or plasma of both APS patients and non-APS controls (4); studies that provided details on the methodology used for anti-β2GPI-D1 testing, including the cut-off values (5); studies that presented sufficient data to calculate the sensitivity and specificity for APS diagnosis.

The inclusion criteria for the meta-analysis investigating the risk of thrombosis associated with anti-β2GPI-D1 were as follows (1): prospective studies that evaluated thrombosis in patients based on their anti-β2GPI-D1 status (2); studies that provided sufficient data to evaluate the risk ratios (RR) of thrombosis associated with anti-β2GPI-D1, or alternatively, time-to-event outcomes expressed as hazard ratios (HR).

The exclusion criteria were (1): non-original studies (2); studies not published in English (3); studies with a small sample size (n < 10) (4); duplicate data from overlapping cohorts (5); studies on pediatric populations.

### Data extraction

Two investigators (LL and JC) independently extracted relevant data using standardized forms, including the first author’s name, year of publication, country, study design, reference standard for APS patients, number of patients and controls, assay for anti-β2GPI-D1, antibody isotype, cut-off values, and number of true positives (TP), false positives (FP), false negatives (FN), and true negatives (TN). For studies enrolling participants from overlapping cohorts, only the data from the study with the highest number of patients was included.

For the analysis of thrombotic risk associated with anti-β2GPI-D1, we extracted the following data: first author’s name, year of publication, country, study design, number of participants and enrollment criteria, baseline age, gender distribution, anti-β2GPI-D1 assay, isotype, cut-off values, length of follow-up, and risk estimates. If the risk estimates (RR) were not reported in the articles, they were calculated based on the available data. Thrombosis was defined as arterial, venous, or small vessel thrombosis in any tissue or organ according to the Sydney Criteria (28).

### Quality assessment

The quality of the studies was assessed using the Quality Assessment Tool for Diagnostic Accuracy Studies version 2 (QUADAS-2) checklist (30) and the Newcastle-Ottawa Scale (NOS) (31). Two investigators (LL and JC) independently evaluated all included studies, with any disagreements resolved through consensus.

### Data synthesis and statistical analysis

The diagnostic accuracy meta-analysis was conducted using the bivariate random-effects regression models to estimate pooled sensitivity, specificity, positive likelihood ratio (PLR), negative likelihood ratio (NLR), and diagnostic odds ratio (DOR). Data extracted from the original studies were organized into diagnostic 2×2 tables (true positives, false positives, true negatives, and false negatives). Missing data were derived from the available information. The pooled sensitivity and specificity, along with their 95% confidence intervals (CIs), were calculated and displayed using forest plots. The hierarchical summary receiver operating characteristic (HSROC) curve was generated to summarize the overall test performance across different thresholds, as recommended in the Cochrane Handbook for Systematic Reviews of Diagnostic Test Accuracy (32). Heterogeneity was assessed by visually inspecting the 95% prediction region in the HSROC curve.

The meta-analysis assessing the risk of thrombosis associated with anti-β2GPI-D1 was performed using RR or HR with their 95% CIs. RRs were calculated if not provided in the original articles. The extracted data were then combined using a random-effects model. Heterogeneity among studies was assessed using the I^2^ statistic (low = 25.0%; moderate = 50.0%; high = 75.0%) (33).

If the meta-analysis included a minimum of 10 studies, subgroup analysis was performed to identify factors contributing to heterogeneity. Meta-regression was conducted to determine whether age and sex influenced the pooled thrombotic risk associated with anti-β2GPI-D1. Potential publication bias was assessed using Deeks’ funnel plot asymmetry test, as recommended by the Cochrane Handbook. All analyses were conducted using Stata (version 16.0), with a p-value < 0.05 considered significant for all tests.

## Results

### Study selection

**Figure 2:**
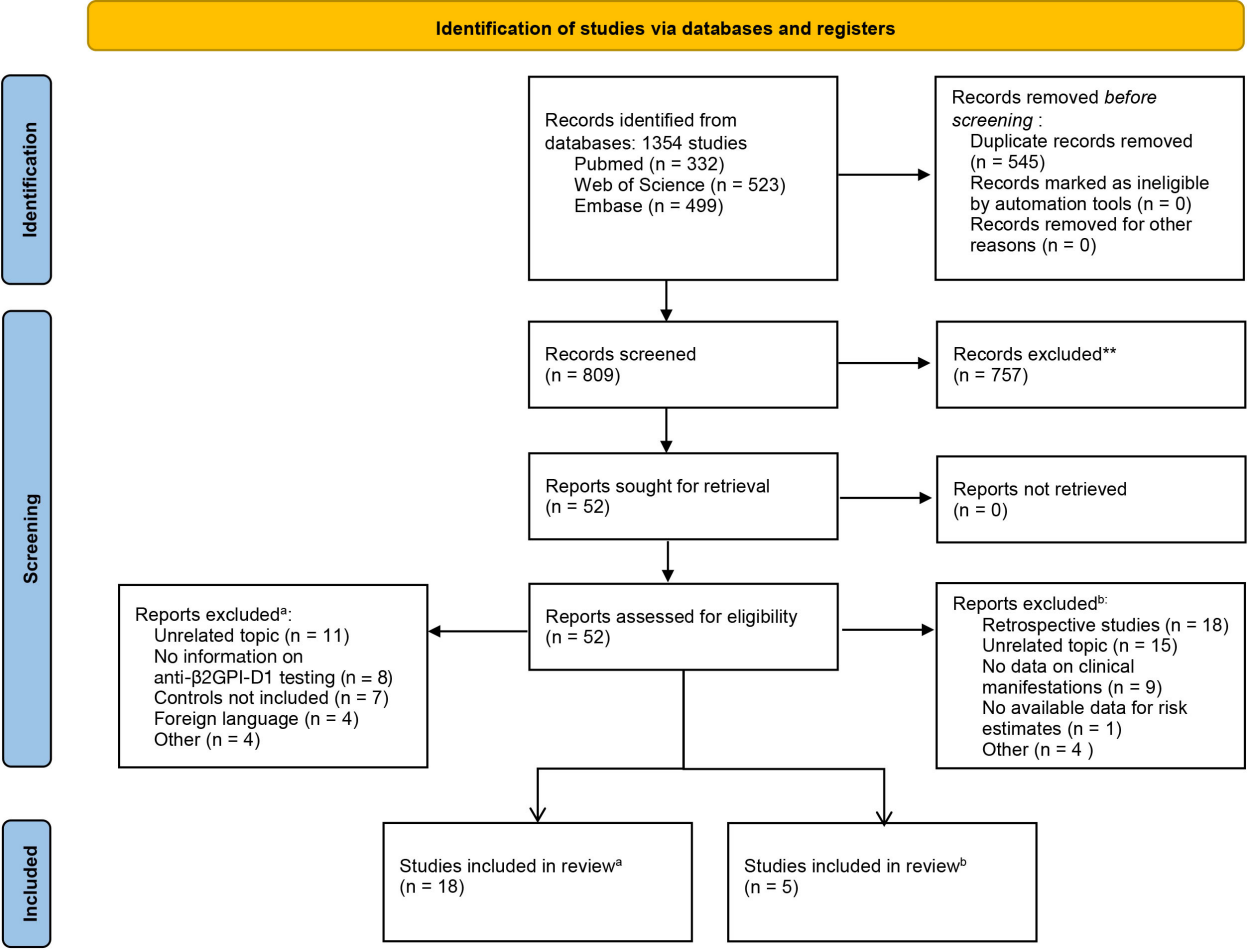
PRISMA flow diagram of the study selection process for the meta-analysis of diagnostic accuracy (a) and thrombotic risk (b).

A total of 1354 publications were initially identified through a comprehensive search. After removing duplicates, 809 studies were screened based on title and abstract. Of these, 52 studies underwent full-text screening to assess eligibility. Ultimately, 18 studies were included in the diagnostic accuracy meta-analysis, and five studies were included in the meta-analysis assessing the risk of thrombosis associated with anti-β2GPI-D1. The study selection process is shown in **Figure 2**.

### Diagnostic accuracy of anti-β2GPI-D1 in APS

Eighteen studies involving 2,060 APS patients and 3,013 controls were included (**Table 1**). All APS patients were diagnosed based on the Sydney Criteria. The control group consisted of 1,667 disease controls, 205 aPL carriers, and 771 healthy controls, except for one study (34) that reported only the total number of controls without specifying the numbers of disease controls and healthy controls. All studies investigated the presence of immunoglobulin G (IgG) isotype. Additionally, one study (35) also investigated the presence of IgM and IgA anti-β2GPI-D1 antibodies. Therefore, we focused on evaluating the diagnostic accuracy of IgG anti-β2GPI-D1. Fifteen studies reported the performance of chemiluminescent immunoassay (CIA), and 3 studies reported enzyme-linked immunosorbent assay (ELISA). Of the 15 studies using CIA, 12 applied a cut-off of 20 chemiluminescence units (CU), two used the 99th percentile of the healthy controls, and one applied a cut-off of 19 CU. Regarding the studies using ELISA, one study used the 99th percentile of the healthy controls, another used the 95th percentile, and one applied a cut-off of mean + 10 standard deviations (SD) of the healthy controls.

**Table 1:** Characteristics of the studies included in the diagnostic meta-analysis.

Author, Year	Country	Study design	APS	Reference standard	Total controls	Disease controls	Healthy controls	Asymptomatic aPL carriers	Assay	Tested isotype	Cut-off	TP	FP	FN	TN
Zhou, 2023 (33)	China	prospective	169	Sydney criteria	209	209 SLE	/	/	CIA (Inova)	IgG	20 CU	55	17	114	192
Reshetnyak, 2023 (34)	Russia	retrospective	111	Sydney criteria	225	64 SLE; 12 probable APS; 7 thrombosis without aPL; 10 RA; 15 Behçet’s disease; 12 SSc; 2 polymyositis; 1 Burger’s endarteritis	102	/	CIA (Inova)	IgG	19 CU	79	24	32	201
Chighizola, 2023 (15)	UK	prospective	171	Sydney criteria	59	/	/	59	CIA (Inova)	IgG	20 CU	109	26	62	33
Liu, 2020 (35)	China	retrospective	192	Sydney criteria	403	103 SLE; 29 SS; 31 RA; 30 AS; 90 SNAPS	120	/	CIA (Inova)	IgG	20 CU	119	23	73	380
Heikal, 2019 (36)	U.S.	retrospective	71	Sydney criteria	145	64 autoimmune disease; 81 other diseases	/	/	CIA (Inova)	IgG	20 CU	23	8	47	135
Nakamura, 2018 (18)	Japan	retrospective	51	Sydney criteria	105	37 SLE; 33 RA; 7 SS; 6 SSc; 4 polymyositis; 2 Behçet’s disease; 2 vasculitis syndrome; 14 others	/	/	CIA (Inova)	IgG	20 CU	31	0	20	106
Litvinova, 2018 (37)	France	prospective	41	Sydney criteria	76	17 SNAPS; 18 thrombotic/obstetrical events	30	11	CIA (Inova)	IgG	20CU	21	1	20	75
Chighizola, 2018 (14)	Italy	retrospective	108	Sydney criteria	27	/	/	27	CIA (Inova)	IgG	20 CU	68	10	40	17
Iwaniec, 2017 (38)	Poland	retrospective	103	Sydney criteria	99	99 SLE	/	/	CIA (Inova)	IgG	HC99% (13.8CU)	64	15	39	84
Zhang, 2016 (39)	China	retrospective	86	Sydney criteria	143	30 non-APS thrombosis; 32 non-APS PRM; 42 SLE	39	/	CIA (Inova)	IgG	20 CU	40	3	46	140
Pericleous, 2016 (40)	UK	retrospective	111	Sydney criteria	319	119 SLE	200	/	ELISA (in house)	IgG IgM IgA	HC99% (10GDIU)	45	15	66	304
Oku, 2016 (19)	Japan	retrospective	61	Sydney criteria	150	37 SLE; 24 RA; 7 scleroderma; 4 myositis; 6 vasculitis syndrome; 5 SS; 7 other autoimmune diseases; 16 non-autoimmune diseases; 34 hepatitis	10	/	CIA (Inova)	IgG	20 CU	32	0	29	150
Mahler, 2016 (31)	UK	retrospective	106	Sydney criteria	272	N.S.	N.S.	/	CIA (Inova)	IgG	20 CU	27	1	79	271
De Craemer, 2016 (16)	Belgium	retrospective	101	Sydney criteria	325	70 SLE; 35 SSc; 18 other autoimmune diseases; 82 DCs	120	/	CIA (Inova)	IgG	20 CU	54	7	47	318
Meneghel, 2015 (32)	Italy	retrospective	88	Sydney criteria	229	11 SLE; 10 SS; 7 polymyositis; 10 SSc; 6 RA; 2 spondyloarthritis; 63 SNAPS	120	/	CIA (IL)	IgG	HC99% (7.1 CU)	48	5	40	224
Andreoli, 2015 (41)	Italy	retrospective	87	Sydney criteria	72	42 systemic autoimmune rheumatic diseases	/	30	Elisa (Inova)	IgG	HC95% (15 AU)	61	44	26	28
Mondejar, 2014 (42)	Spain	retrospective	39	Sydney criteria	77	30 RA; 17 other rheumatological diseases	30	/	CIA (Inova)	IgG	20 CU	14	2	25	75
De Laat, 2009 (43)	Netherlands	retrospective	364	Sydney criteria	78	/	/	78	ELISA (In-house)	IgG	mean+10SD (HC)	218	25	146	53

APS, antiphospholipid syndrome; HC, healthy control; DC, disease control; HC99%, the 99th percentile value of the healthy controls; HC95%, the 95th percentile value of the healthy controls; CIA, chemiluminescent immunoassay; CU, chemiluminescence units; ELISA, enzyme-linked immunosorbent assay; aPL, antiphospholipid antibody; SLE, systemic lupus erythematosus; RA, rheumatoid arthritis; SSc, systemic sclerosis; SS, Sjögren’s syndrome; AS, ankylosing spondylitis; SNAPS, seronegative antiphospholipid syndrome; PRM, pregnancy-related morbidity; N.S., not specified; mean+10SD, mean value plus 10 standard deviations of the healthy controls.

### Diagnostic accuracy of anti-β2GPI-D1 in APS and all controls

**Figure 3:**
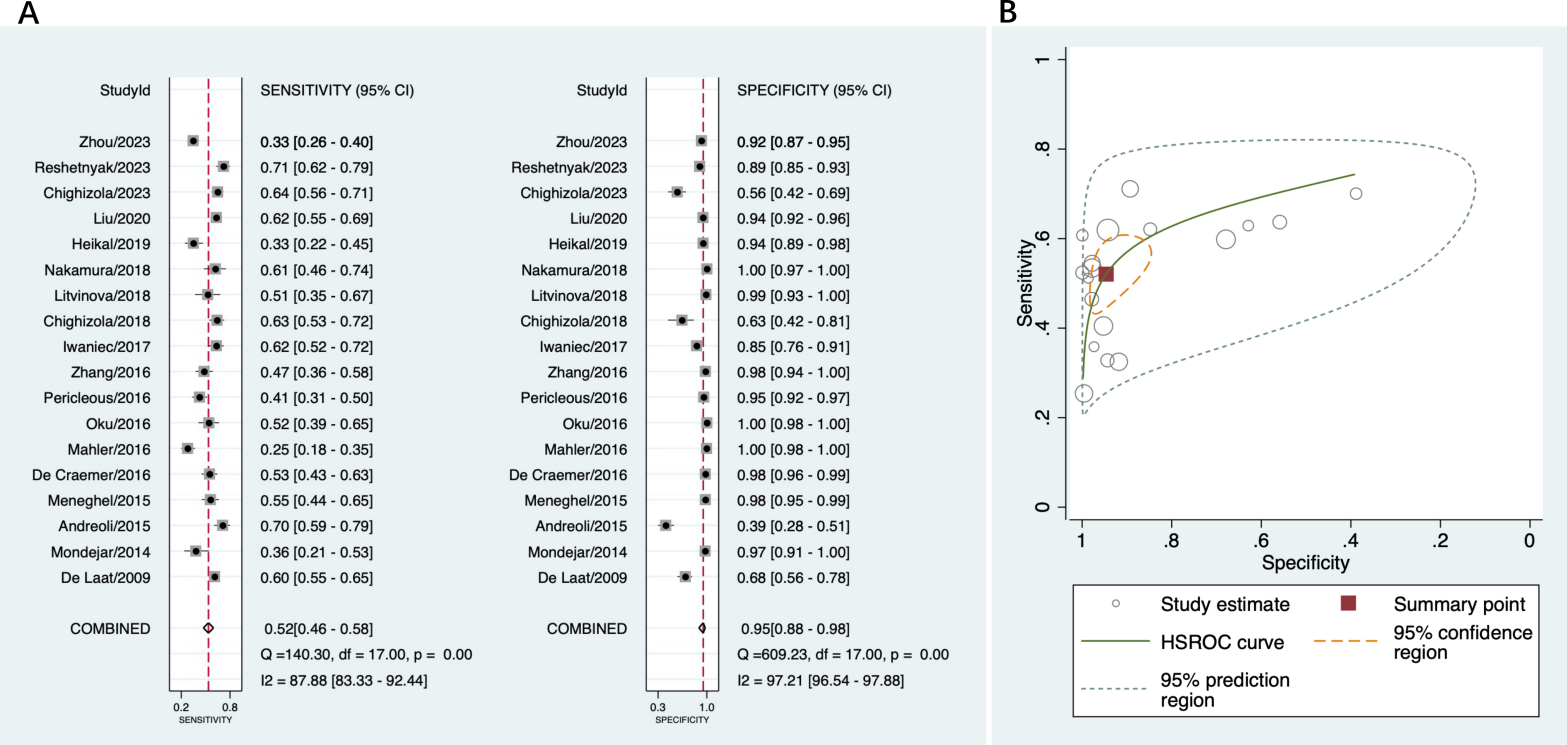
Diagnostic accuracy of anti-β2GPI-D1 in APS and all controls. **(A)** forest plot of pooled sensitivity and specificity; **(B)** HSROC.

A total of 18 studies were included in this part of the meta-analysis (14, 15, 18, 36–43). The sensitivity and specificity data from each study, along with the summary estimates, are presented as forest plots in **Figure 3A**. The sensitivity of anti-β2GPI-D1 ranged between 25% and 71%, and its specificity ranged from 39% to 100%. The pooled sensitivity of anti-β2GPI-D1 was 52% (95% CI 46%-58%), and the pooled specificity was 95% (95% CI 88%-98%). The pooled PLR was 9.7 (95% CI 4.6-20.5), and the pooled NLR was 0.51 (95% CI 0.45-0.57). Additionally, the pooled DOR was 19 (95% CI 9-41). The HSROC curve summarizing the results from all included studies is shown in **Figure 3B**. The 95% prediction region showed that high heterogeneity remained among these studies.

### Diagnostic accuracy of anti-β2GPI-D1 in APS and disease controls

**Figure 4:**
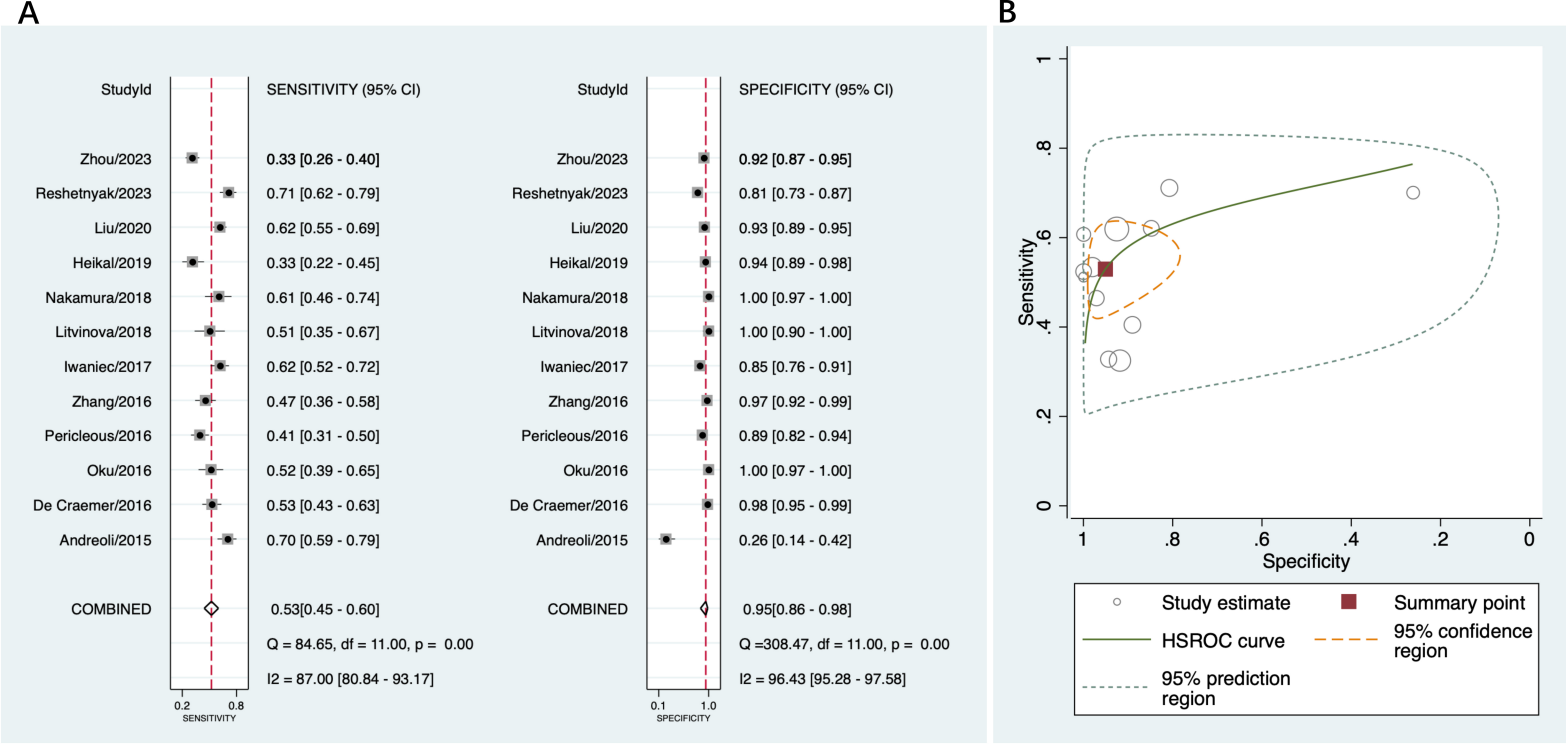
Diagnostic accuracy of anti-β2GPI-D1 in APS and disease controls. **(A)** forest plot of pooled sensitivity and specificity; **(B)** HSROC.

A total of 12 studies that provided diagnostic accuracy data based on disease controls were included in this part (16, 18, 19, 36–44). The disease controls consisted of patients with suspected APS, hepatitis, infectious diseases, systemic lupus erythematosus, rheumatoid arthritis, systemic sclerosis, Sjögren’s syndrome, Behçet’s disease, and other autoimmune disorders. Among these studies, the sensitivity of anti-β2GPI-D1 varied from 33% to 71%, and the specificity varied from 26% to 100%. The pooled sensitivity of anti-β2GPI-D1 was 53% (95% CI 45%-60%), and the pooled specificity was 95% (95% CI 86%-98%) (**Figure 4A**). The pooled PLR was 10.8 (95% CI 3.7-31.2), the pooled NLR was 0.49 (95% CI 0.42-0.58), and the pooled DOR was 22 (95% CI 7-66). As shown in **Figure 4B**, high heterogeneity was observed among these studies.

### Diagnostic accuracy of anti-β2GPI-D1 in APS and healthy controls

**Figure 5:**
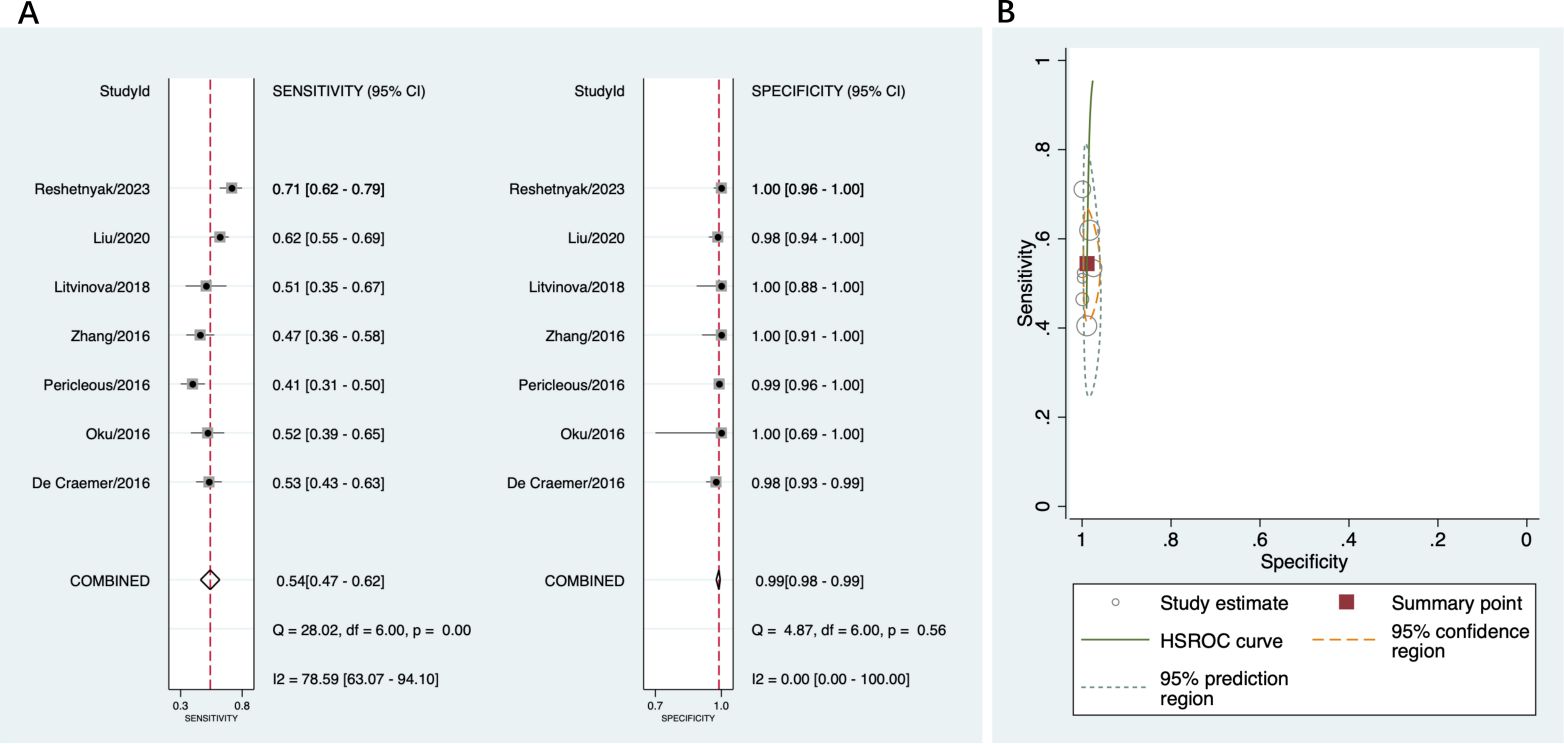
Diagnostic accuracy of anti-β2GPI-D1 in APS and healthy controls. **(A)** forest plot of pooled sensitivity and specificity; **(B)** HSROC.

In seven studies included in this part (16, 19, 37, 38, 40, 42, 43), the sensitivity ranged between 41% and 71% across the studies, and the specificity ranged from 98% to 100%. The pooled sensitivity and specificity was 54% (95% CI 47%-62%) and 99% (95% CI 98%-99%), respectively (**Figure 5A**). The pooled PLR was 48.7 (95% CI 22.4-106.0) and pooled NLR was 0.46 (95% CI 0.39-0.54); the pooled DOR was 106 (95% CI 45-246). As shown in **Figure 5B**, there was high heterogeneity in sensitivity and low heterogeneity in specificity among these studies.

### Diagnostic accuracy of anti-β2GPI-D1 in APS and asymptomatic aPL carriers

**Figure 6:**
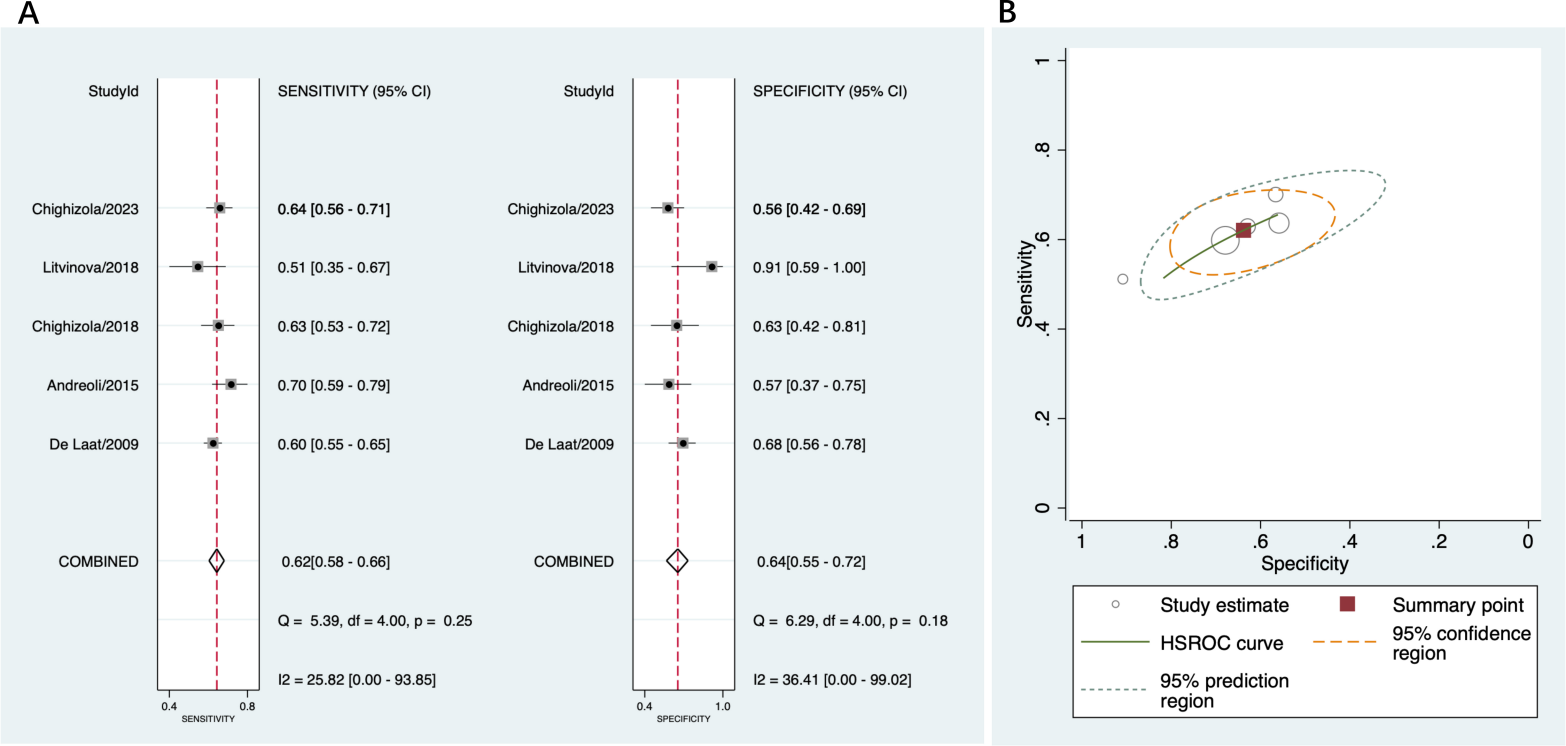
Diagnostic accuracy of anti-β2GPI-D1 in APS and asymptomatic aPL carriers. **(A)** forest plot of pooled sensitivity and specificity; **(B)** HSROC.

A total of five studies were included in this part (14, 15, 40, 44, 45). Across the studies, the sensitivity ranged from 51% to 70%, and the specificity ranged from 57% to 91%. The pooled sensitivity was 62% (95% CI 58%-66%) and the pooled specificity was 64% (95% CI 55%-72%) (**Figure 6A**). The pooled PLR was 1.7 (95% CI 1.4-2.1) and pooled NLR was 0.59 (95% CI 0.52-0.68); the pooled DOR was 3 (95% CI 2-4). **Figure 6B** indicated low heterogeneity in sensitivity and moderate heterogeneity in specificity among these studies.

### Risk of thrombosis associated with anti-β2GPI-D1

**Figure 7:**
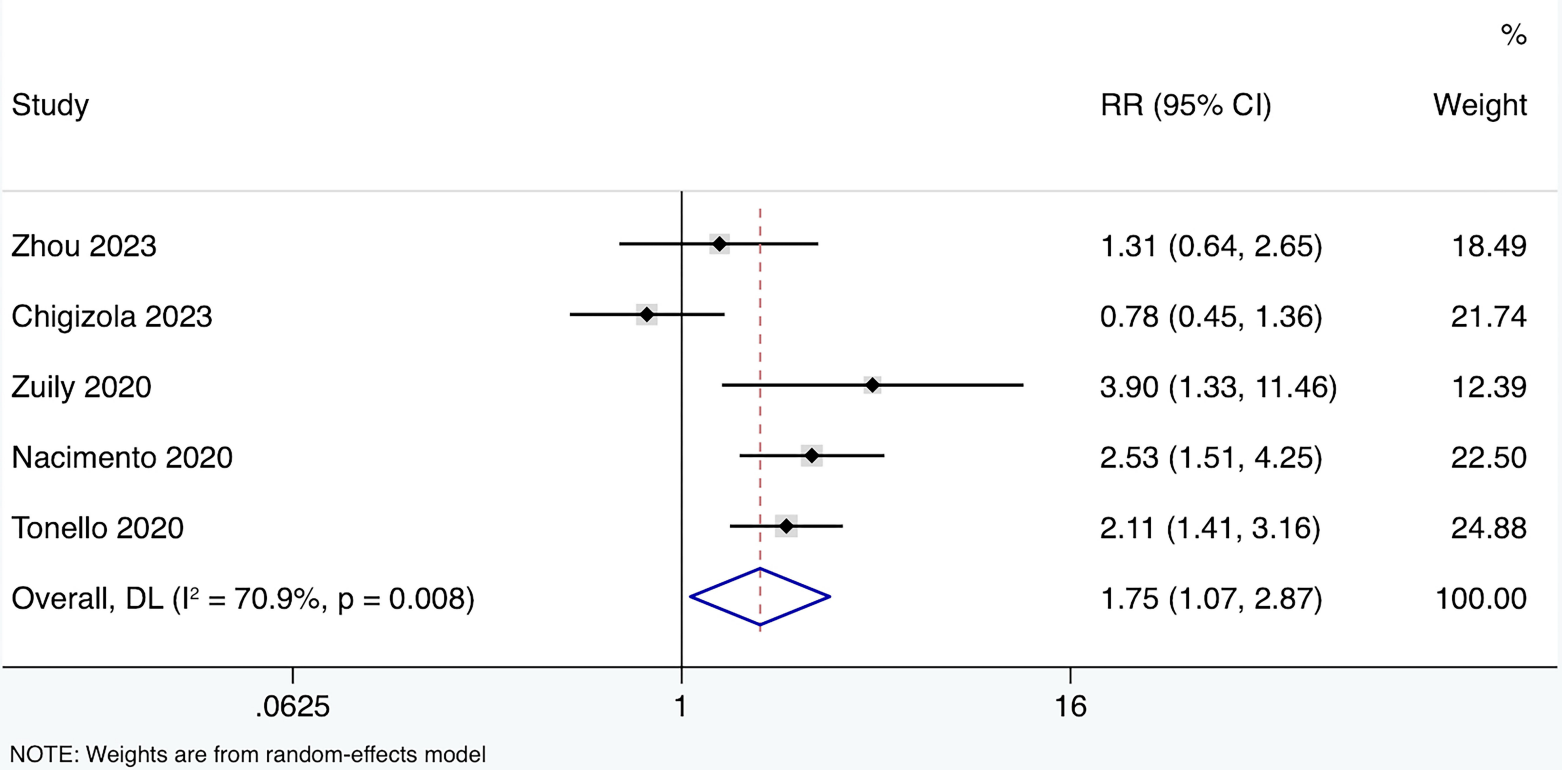
Forest plot showing the risk of thrombosis associated with anti-β2GPI-D1. RR, risk ratio.

A total of five prospective cohort studies (15, 36, 46–48) were included, involving 210 APS patients, 430 aPL carriers, and 42 SLE patients (**Table 2**). All studies reported the performance of CIA in detecting the IgG isotype. Four out of the five studies used a positivity cut-off of 20 CU, while one study used the 99th percentile of healthy controls as the cut-off. The average follow-up period ranged from 25 to 82.2 months. The HR for the effect of anti-β2GPI-D1 on thrombosis risk was reported in one study, and the RR could be estimated in the remaining four studies. Among these four studies, a total of 41 thrombotic events were observed. When these results were combined, the overall risk of thrombosis in anti-β2GPI-D1 positive patients was significantly higher compared to anti-β2GPI-D1 negative patients (RR 1.75 95%CI 1.07-2.87) (**Figure 7**). Moderate heterogeneity was detected among these studies (I^2^ = 70.9%, p<0.01). Meta-regression showed that age and sex did not have a significant effect on the pooled risk ratio of thrombosis associated with anti-β2GPI-D1 (p=0.36). Meta-regression indicated that age and sex did not have a significant effect on the pooled risk ratio of thrombosis associated with anti-β2GPI-D1 (p=0.64 and 0.20, respectively).

**Table 2:** Characteristics of the studies included in the meta-analysis of thrombosis risk associated with anti-β2GPI-D1.

Author, year	Country	Design	Number of participants and enrollment criteria	Baseline age (years)	Gender (F/M)	Assay	Isotype	Cut-off	Length of follow-up	Risk estimates
Zhou, 2023 (33)	China	prospective, cohort	169 APS	34 (31, 41)	117/52	CIA(Inova)	IgG	20 CU	25 (21-34) months	RR* 1.31 (0.64-2.65)
Chighizola, 2023 (15)	UK	prospective, cohort	230 aPL carriers	45.0 ± 12.7	159/71	CIA(Inova)	IgG	20 CU	every 12 ± 3 months for 3 years	RR* 0.78 (0.45-1.36)
Zuily, 2020 (44)	France	prospective, cohort	95 aPL carriers 42 SLE	43.5 ± 15.4	107/30	CIA(Inova)	IgG	20 CU	43.1 ± 20.7 months	HR** 3.90 (1.33–11.46)
Nascimento, 2020 (45)	Brazil	prospective, cohort	41 APS	43 ± 10	39/5	CIA(Inova)	IgG	HC99%	39 (9-46) months	RR* 2.53 (1.51-4.25)
Tonello, 2018 (46)	Italy	prospective, cohort	105 aPL carriers	44.6 ± 10.7	96/9	CIA(Inova)	IgG	20CU	82.2 ± 46.7 months	RR* 2.11 (1.41–3.16)

APS, antiphospholipid syndrome; aPL, antiphospholipid antibody; CIA, chemiluminescent immunoassay; CU, chemiluminescence units; HC99%, the 99th percentile value of the healthy controls.

*derived from the data provided in the original studies; **multvariate adjusted.

### Quality assessment

The quality assessment of diagnostic studies was conducted according to the QUADAS2, including evaluations of both risk of bias and applicability concerns (**Supplementary Figure S1**). All included studies exhibited low applicability concerns. In terms of risk of bias, 77.8% of studies showed a high risk in the patient selection domain, 16.7% showed low risk, and 5.6% had unclear risk. The most common reason for assigning a high risk was that the sample of patients was not selected consecutively or randomly. For the index test domain, 11.1% of studies showed low risk, while 88.9% had unclear risk, primarily due to the lack of reporting on whether the reference standard was known during the index test. For the reference standard and flow and timing domains, all included studies demonstrated low risk.

Regarding the meta-analysis of the risk of thrombosis, based on the NOS assessment, two studies were considered high quality, and three studies were considered moderate quality (**Supplementary Table S1**). Most studies were downgraded due to the failure to adjust for potential confounders and an inadequate follow-up period.

### Heterogeneity analysis and publication bias

**Figure 8:**
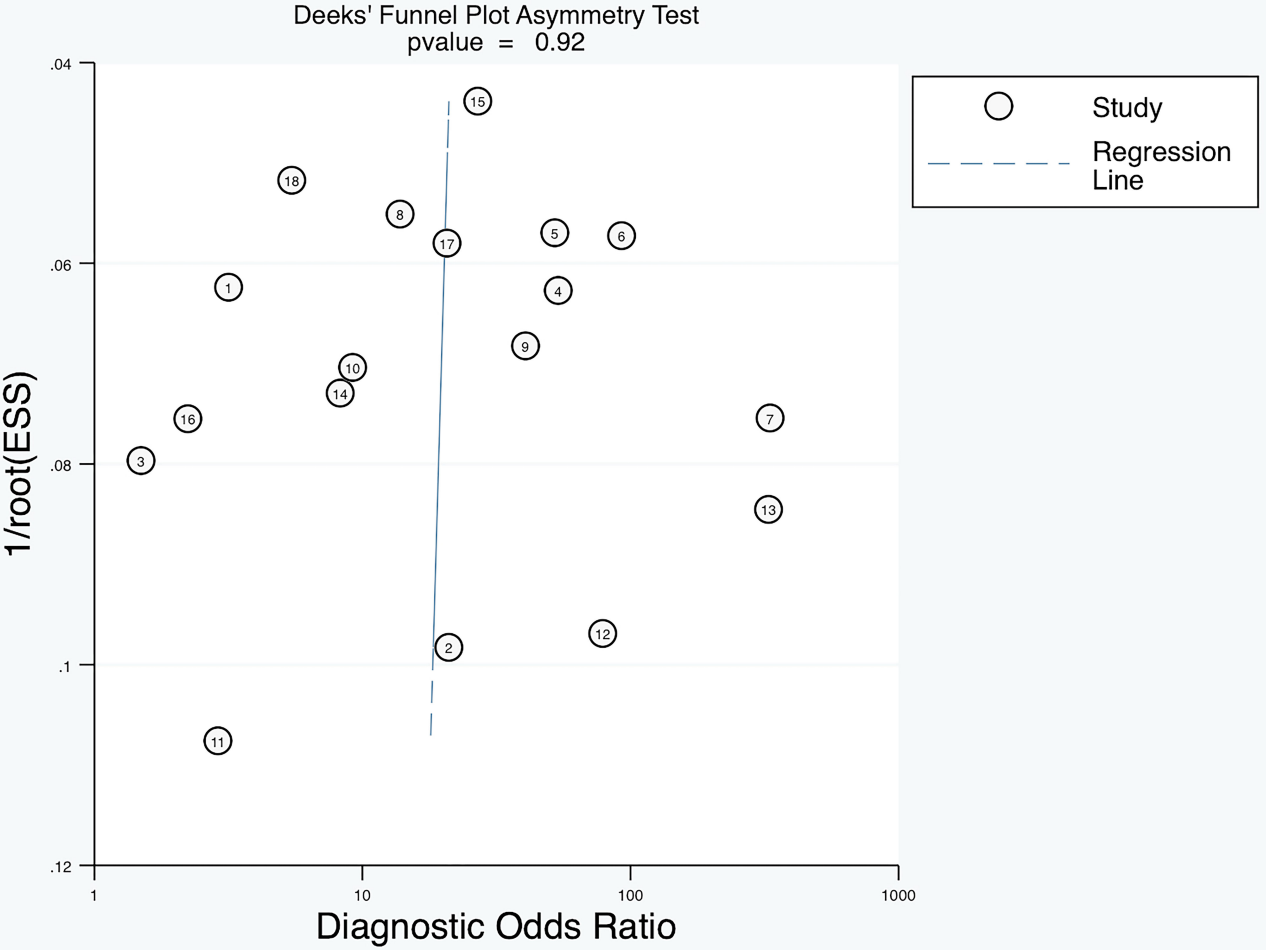
Deeks’ funnel plot asymmetry test showing no significant publication bias of diagnostic meta-analysis (p=0.92).

To explore the heterogeneity in sensitivity and specificity, subgroup analyses were conducted based on study design, cut-off values, assay methods, and sample size (**Table 3**). The results showed that detecting anti-β2GPI-D1 using CIA demonstrated significantly higher specificity (96% [95% CI 93%-99%]) compared to ELISA (75% [95% CI 43%-100%], p = 0.04). However, no significant differences in sensitivity were observed across the subgroups. Deeks’ funnel plot asymmetry test did not reveal significant publication bias (p = 0.92) (**Figure 8**).

**Table 3:** Subgroup analysis of diagnostic accuracy of anti-β2GPI-D1 in APS.

Characteristic	No. of studies	Pooled sensitivity (95%CI)	P value	Pooled specificity (95%CI)	P value
Assay					
CIA	15	51% (44%-58%)	0.43	96% (93%-99%)	**0.04**
ELISA	3	57% (42%-72%)		75% (43%-100%)	
Cut-off					
Provided by the manufacturer	13	50% (42%-57%)	0.28	96% (93%-100%)	0.20
Determined from HCs	5	58% (46%-69%)		85% (68%-100%)	
Study design					
prospective	3	48% (32%-63%)	0.68	90% (72%-100%)	0.64
retrospective	15	53% (46%-60%)		95% (91%-99%)	
Sample size					
>200	13	50% (43%-58%)	0.37	95% (91%-100%)	0.76
≤200	5	57% (44%-69%)		93% (83%-100%)	

CIA, chemiluminescent immunoassay; ELISA, enzyme-linked immunosorbent assay; HC, healthy control.

Regarding the meta-analysis on the risk of thrombosis, moderate to high heterogeneity was identified among the studies (I² = 70.9%, p < 0.01). However, subgroup analysis could not be performed due to the limited number of studies. Additionally, publication bias could not be assessed as fewer than ten studies were included.

## Discussion

This is the first study to comprehensively review all available and relevant articles and assess the overall diagnostic accuracy of anti-β2GPI-D1 for APS. This systematic review and meta-analysis included 18 studies with a total of 2,060 APS patients and 3,013 controls from various countries worldwide. The results showed that anti-β2GPI-D1 has a high specificity of 95% (95% CI 88%-98%) and a moderate sensitivity of 52% (95% CI 46%-58%), indicating that anti-β2GPI-D1 is a potential marker for diagnosing APS, particularly beneficial in confirming the diagnosis due to its high specificity. Notably, anti-β2GPI-D1 demonstrated higher specificity for disease controls (95% [95% CI 86%-98%]) and healthy controls (99% [95% CI 98%-99%]) compared to aPL carriers (64% [95% CI 55%-72%]). This suggests that anti-β2GPI-D1 may provide greater diagnostic value in distinguishing APS from other autoimmune diseases and healthy individuals, while its diagnostic utility in differentiating APS from aPL carriers may be limited.

Our study further explored the reasons for heterogeneity through subgroup analysis and identified the assay method as one of the main contributors to heterogeneity in specificity. The quality and variability of assay methods are common factors that can significantly impact the specificity of a biomarker, potentially leading to inconsistencies in results across different studies. For example, when detecting anti-β2GPI-D1 using ELISA, the charge of the solid-phase surface used to immobilize β2GPI can affect the exposure of the G40-R43 epitope (49). This change may result in differences in antibody binding and, consequently, variations in the results. Andreoli et al. (44) reported a specificity of 39% (95% CI 28%-51%) using ELISA, suggesting that the use of this assay may have contributed to the low specificity observed in their study. As we included all these studies for a comprehensive review, a comparative analysis of the diagnostic accuracy between ELISA and CIA could be beneficial for future research.

The diagnosis of APS is based on a combination of clinical features and the detection of antiphospholipid antibodies, including LA, aCL, and anti-β2GPI antibodies. However, a broader group of “non-criteria” antibodies targeting various antigens are also found in APS patients and may contribute to the pathogenesis of the disease (50, 51). Among these, anti-β2GPI-D1 has received considerable attention due to strong evidence from animal and clinical studies indicating its role in increasing the risk of thrombotic complications (52, 53). Notably, there is no evidence that any domain other than domain 1 is involved in mediating these thrombotic events (54). This is supported by previous studies showing that antibodies targeting domains 4/5 in plasma are not associated with clinical manifestations of APS (14, 20). Moreover, anti-domain 5 antibodies did not induce thrombus formation or vascular occlusion in LPS-treated rats, likely due to their inability to interact with cell-bound β2GPI (55).

Given this, testing for anti-β2GPI-D1 has been proposed as an additional diagnostic tool, particularly in patients with suspected APS when routine anti-β2GPI tests yield negative results (54). Previous studies have reported a positivity rate for anti-β2GPI-D1 in seronegative APS patients ranging from absent or low (<5%) to as high as 16% (35, 38, 40, 56, 57), supporting its potential utility in this subset of patients. A previous systematic review (58) evaluated studies from 1986 to 2016 and reported an overall prevalence of anti-β2GPI-D1 in APS at 45.4%. In our meta-analysis, which included all studies published thereafter, we confirmed a pooled sensitivity for anti-β2GPI-D1 of 52% (95% CI 46%-58%) in a more extensive study population. Based on our results, anti-β2GPI-D1 may play a significant role in the evaluation of seronegative APS by providing additional serologic information, potentially leading to a revised diagnosis of APS.

In addition, this systematic review and meta-analysis identified a higher risk of thrombosis associated with anti-β2GPI-D1, based on data from 5 prospective cohort studies. These studies included 210 APS patients, 430 aPL carriers, and 42 SLE patients, and were assessed to be of moderate to high quality. Meta-regression analysis revealed that confounding factors such as age and sex did not significantly affect the risk of thrombosis associated with anti-β2GPI-D1. Our results suggest that anti-β2GPI-D1 may serve as a predictor of thrombosis and contribute to the risk stratification of patients with APS. Consistent with these results, previous retrospective analyses also reported an increased thrombotic risk associated with anti-β2GPI-D1 (pooled odds ratio 1.99 [95% CI 1.52–2.6]) (58). Moreover, additional evidence underscores the importance of anti-β2GPI-D1 in thrombotic risk stratification. For instance, anti-β2GPI-D1 is more frequent and at higher titers in APS patients with triple aPL positivity, a recognized hallmark of elevated thrombotic risk (16, 56, 59). Furthermore, the presence and titers of anti-β2GPI-D1 have also been associated with the Global Antiphospholipid Syndrome Score (GAPSS), a validated risk-scoring system in APS (17, 47).

This meta-analysis has several strengths. The primary strength lies in the rigorous statistical methods we employed to assess diagnostic accuracy, including bivariate random-effects regression models and the HSROC curve. Additionally, the inclusion of prospective cohort studies enhances the reliability of our findings and provides a more comprehensive evaluation of the association between anti-β2GPI-D1 and thrombosis. However, some limitations should be noted. First, we identified high heterogeneity across studies in the diagnostic accuracy estimates, though such variability is often expected in meta-analyses of diagnostic tests. To address this, we explored potential sources of this heterogeneity through subgroup analysis, which suggested that the assay method used for anti-β2GPI-D1 may have contributed, at least in part, to this variability. In addition, moderate heterogeneity was observed in the pooled risk estimates for thrombosis associated with anti-β2GPI-D1; however, due to the limited number of available studies, further investigation into the sources of this heterogeneity was not possible. Second, only one of the included studies provided multivariate-adjusted risk estimates for thrombosis associated with anti-β2GPI-D1. Therefore, a meta-regression analysis was then undertaken to account for potential confounding factors such as age and sex differences across studies, revealing that these factors did not significantly influence the pooled risk estimates. Additionally, most studies reported similar baseline characteristics for thrombosis risk factors across the study groups. While this does not entirely rule out the possibility of confounding, the similarity in these baseline characteristics strongly suggests that the observed thrombosis is primarily associated with the presence of anti-β2GPI-D1. Further research with multivariate-adjusted analyses, particularly in larger populations with homogeneous clinical characteristics, is needed to validate the predictive value of anti-β2GPI-D1 in APS.

## Conclusions

In conclusion, our meta-analysis demonstrates that anti-β2GPI-D1 offers good diagnostic accuracy with high specificity. It has significant value in distinguishing APS from other autoimmune diseases and may also provide additional diagnostic information for specific patient populations, such as those with seronegative APS. Furthermore, our results indicate that anti-β2GPI-D1 has a strong predictive value for identifying patients at risk of developing thrombosis, making it a potentially valuable tool for risk stratification in APS patients. Further prospective studies with larger sample sizes and homogeneous clinical characteristics are needed to confirm our findings.

## Data Availability

The data analyzed in this study is subject to the following licenses/restrictions: The dataset is restricted due to privacy concerns and is available upon request from the corresponding author. Requests to access these datasets should be directed to jiangyongmei_1@163.com.

## Supplementary material

Supplementary Figure 1 Quality assessment of included studies on the diagnostic accuracy of anti-β2GPI-D1 by QUADAS-2 in each study **(A)** and in summary **(B)**.
