# Supplementary figures and images for: Anti-β2 glycoprotein domain 1 antibody as a diagnostic marker for antiphospholipid syndrome and a predictor of thrombosis: a systematic review and meta-analysis
Source: Front Immunol. 2025 Apr 23;16:1541165. doi: 10.3389/fimmu.2025.1541165 (PMC12056313; doi:10.3389/fimmu.2025.1541165)

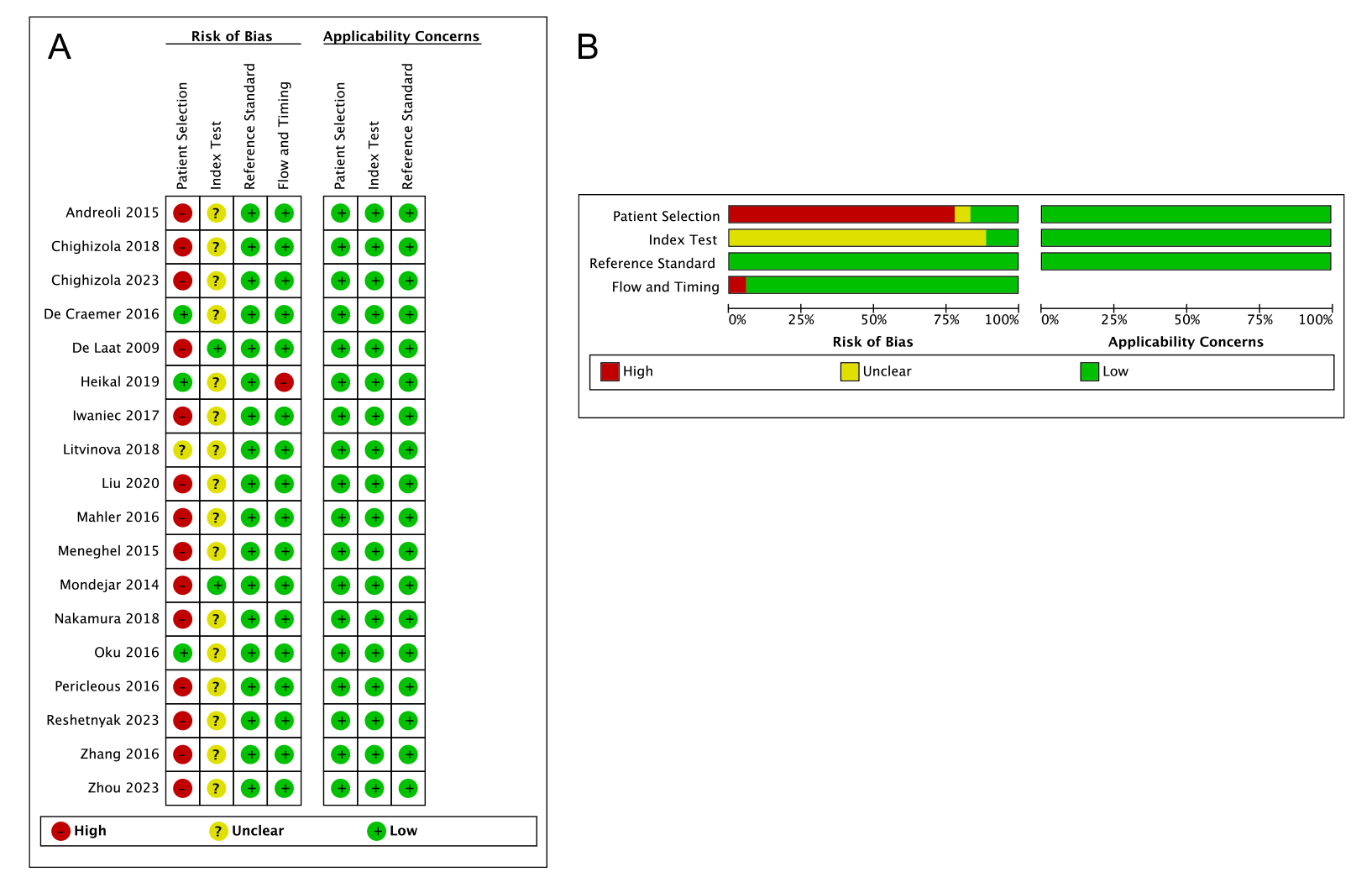

Supplement: Supplementary Figure 1 — Quality assessment of included studies on the diagnostic accuracy of anti-β2GPI-D1 by QUADAS-2 in each study (A) and in summary (B). [file Image1.tif]
