# Supplementary material for: Anti-β2 glycoprotein domain 1 antibody as a diagnostic marker for antiphospholipid syndrome and a predictor of thrombosis: a systematic review and meta-analysis
Source: Front Immunol. 2025 Apr 23;16:1541165. doi: 10.3389/fimmu.2025.1541165 (PMC12056313; doi:10.3389/fimmu.2025.1541165)
Supplement: Supplementary file 2 [file Table1.docx]

Table S1 Quality assessment of studies on the risk of thrombosis associated with anti-β2GPI-D1 using the Newcastle-Ottawa scale.

| Study | Selection (out of 4) | Comparability(out of 2) | Outcome(out of 3) | Total score |
| --- | --- | --- | --- | --- |
| Zhou, 2023 | 3 | 0 | 2 | 5 |
| Chighizola, 2023 | 4 | 0 | 2 | 6 |
| Zuily, 2020 | 4 | 1 | 2 | 7 |
| Nascimento, 2020 | 4 | 0 | 2 | 6 |
| Tonello, 2018 | 4 | 0 | 3 | 7 |
